# Efficacy of fungal decolorization of a mixture of dyes belonging to different classes

**DOI:** 10.1590/S1517-838246246220140167

**Published:** 2015-06-01

**Authors:** Wioletta Przystas, Ewa Zablocka-Godlewska, Elzbieta Grabinska-Sota

**Affiliations:** Environmental Biotechnology Department, Silesian University of Technology, Gliwice, Poland, Environmental Biotechnology Department, Silesian University of Technology, Gliwice, Poland.

**Keywords:** decolorization, fungal dye removal, dye mixture, zootoxicity, phytotoxicity

## Abstract

Dyes are the most difficult constituents to remove by conventional biological wastewater treatment. Colored wastewater is mainly eliminated by physical and chemical procedures, which are very expensive and have drawbacks. Therefore, the advantage of using biological processes, such as the biotransformation of dyes, is that they may lead to complete mineralization or formation of less toxic products. To prove the possibility of using fungal processes for decolorization and other applications, the analysis of the toxicity of the processes' products is required. The decolorization of the mixture of two dyes from different classes - triphenylmethane brilliant green and azo Evans blue (GB - total concentration 0.08 g/L, proportion 1:1 w/w) - by *Pleurotus ostreatus* (BWPH and MB), *Gloeophyllum odoratum* (DCa), RWP17 (*Polyporus picipes*) and *Fusarium oxysporum* (G1) was studied. Zootoxicity (*Daphnia magna*) and phytotoxicity (*Lemna minor*) changes were estimated at the end of the experiment. The mixture of dyes was significantly removed by all the strains that were tested with 96 h of experimental time. However, differences among strains from the same species (*P. ostreatus*) were noted. Shaking improved the efficacy and rate of the dye removal. In static samples, the removal of the mixture reached more than 51.9% and in shaken samples, more than 79.2%. Tests using the dead biomass of the fungi only adsorbed up to 37% of the dye mixture (strain BWPH), which suggests that the process with the living biomass involves the biotransformation of the dyes. The best results were reached for the MB strain, which removed 90% of the tested mixture under shaking conditions. Regardless of the efficacy of the dye removal, toxicity decreased from class V to class III in tests with *D. magna*. Tests with *L. minor* control samples were classified as class IV, and samples with certain strains were non-toxic. The highest phytotoxicity decrease was noted in shaken samples where the elimination of dye mixture was the best.

## Introduction

Synthetic azo, triphenylmethane and anthraquinone dyes are used in textile, food, cosmetics, papermaking and pharmaceutical industries (Swamy and Ramsay, 1999; Padamavathy *et al.*, 2003). Their complex aromatic structure allows for their resistance to light and water oxidation and biodegradation. It is a feature desired by the industry using the dye, but it is dangerous for the environment. The non-biodegradable nature of most of the dyes causes their accumulation in surface water and sediments, especially at places of wastewater discharge. It reduces aquatic diversity by blocking the passage of sunlight through the water and creates problems for photosynthetic aquatic plants and algae, thus, affecting the ecological balance in the aquatic system. Many synthetic dyes are toxic, mutagenic and carcinogenic (Namasivayam and Sumithra, 2005; Divaniyam *et al.*, 2010). Textile finishing wastewater and house effluents may contain different classes of organic dyes, chemicals and auxiliaries (Lin and Chen, 1997; Dos Santos *et al.*, 2007). They are characterized by a strong color and large amount of suspended solids (Golob *et al.*, 2005). The presence of these dyes in wastewater effluents is highly visible even in small concentrations. Dyes are the most difficult constituents to treat by conventional biological wastewater treatment (Gill *et al.*, 2001; Robinson *et al.*, 2001; Yesilada *et al.*, 2003; Dos Santos *et al.*, 2007; Khan *et al.*, 2013). Effective dye removal is required due to stringent government legislation and strict environmental regulations (Pandey *et al.*, 2007).

Colored wastewater from the textile industry is one of the most serious problems in the world. In some regions, the textile industry is a significant contributor to the economic growth of a country. During normal textile dyeing and finishing, the dye used during operations may be changed from day to day and sometimes even several times a day. Approximately 10,000 different dyes are commercially available. Imperfections in textile dyeing processes cause a 10–15% loss of the applied dyes (Gill *et al.*, 2001; Robinson *et al.*, 2001; Yesilada *et al.*, 2003; Divaniyam *et al.*, 2010). Colored wastewater is mainly eliminated by physical and chemical processes, such as coagulation, flocculation, adsorption, flotation, precipitation, oxidation and reduction, ozonation and membrane separation. However, they are very expensive and have drawbacks (Azmi *et al.*, 1998; Robinson *et al.*, 2001). The most widely used treatment system, conventional activated sludge, poorly removes the most widely used dyes and is clearly ineffective at decolorizing textile effluent, even when mixed and treated together with sewage (Robinson *et al.*, 2001; Al-Kdasi *et al.*, 2004). Environmentally friendly and cost-competitive bioremediation by microorganisms should be improved. Treatment of textile effluent requires a sound and efficient system able to achieve adequate color removal. There are many publications confirming the high potential of bacterial, fungal and algal species in dye removal (Banat *et al.*, 1996; Azmi *et al.*, 1998; Pointing and Vrijmoed, 2000; Fu and Viraraghavan, 2001; Robinson *et al.*, 2001; Sharma *et al.*, 2004; Deng *et al.*, 2008; Przystas *et al.*, 2009; Khan *et al.*, 2013). The removal of dyes is achieved due to biodegradation (mineralization or biotransformation) and/or adsorption on biomass. These processes use low-cost biological materials, as living or dead microorganisms, industrial waste from the breeding of mushrooms, chitosan, peat and plant wastes. The advantage of using biological processes is biotransformation, which may lead to complete mineralization of the dyes or the formation of less toxic products (Azmi *et al.*, 1998; Pointing and Vrijmoed, 2000; Robinson *et al.*, 2001; Deng *et al.*, 2008).

Fungi could be an excellent candidate for dye removal. Most of them use an extracellular enzymatic system that transforms aromatic substances, such as lignin, PAH or pesticides. Much attention is currently focused on fungal decolorization processes. The most widely studied are white rot fungi. They produce non-specific enzymes, such as lignin peroxidase, manganese peroxidase and laccase, which degrade many aromatic compounds. Fungi are used as sorbents and/or enzyme producers involved in biodegradation/biotransformation (Knapp *et al.*, 1995; Azmi *et al.*, 1998; Banat *et al.*, 1996; Swamy and Ramsay, 1999; Pointing and Vrijmoed, 2000; Fu and Viraraghavan, 2001; Gill *et al.*, 2001; Toh *et al.*, 2003; Wesenberg *et al.*, 2003; Padamavathy *et al.*, 2003; Novotny *et al.*, 2004; Forgacs *et al.*, 2004; Dos Santos *et al.*, 2007; Deng *et al.*, 2008; Zablocka-Godlewska *et al.*, 2009; Przystas *et al.*, 2009; Divaniyam *et al.*, 2010; Przystas *et al.*, 2013; Hadibarata *et al.*, 2013; Si *et al.*, 2013).

The main objective of our study is to assess the efficacy of dye mixture decolorization by fungal strains *Pleurotus ostreatus* (BWPH and MB), *Polyporus picipes* (RWP17), *Gloeophyllum odoratum* (DCa) and *Fusarium oxysporum* (G1). Dyes used for the mixture were from different classes, which are not frequently tested during decolorization studies. To test for environmental safety, the zootoxicity and phytotoxicity of the dye mixture, as well as the byproducts produced during the decolorization process, were determined.

## Materials and Methods

### Tested organisms and culture conditions

Fungal strains *Pleurotus ostreatus* (BWPH and MB), *Gloeophyllum odoratum* (DCa), RWP17 (*Polyporus picipes*) and *Fusarium oxysporum* (G1) (bankit1276596 544 bp DNA linear PLN 15-OCT-2009) were isolated by the tissue method (MEA medium (Difco)) from the fruiting bodies of fungi collected in the woods near Gliwice (southern Poland, Upper Silesia). Samples were incubated at 26 °C. Cultures were maintained in MEA slants and stored at 4 °C.

### Decolorization experiment

The aim of this study was to determine the effectiveness of dye mixture decolorization using single strains. The dye concentration for the main experiment was determined experimentally. The influence of five different concentrations (0.02, 0.04, 0.06, 0.08, 0.1 and 0.12 g/L) of Evans blue and brilliant green on the decolorization effectiveness was estimated. The dye mixture was prepared with an equal proportion (1:1 w/w) of both dyes. Tube tests on liquid YEPG medium were performed in triplicate. The absorbance was measured after 7 days of incubation.

Samples in the main experiment were prepared by the addition of two pieces of mycelium (Ø 5 mm) cultured for 7 days on MEA (Fluka Analytical Sigma-Aldrich, India) in Erlenmeyer flasks with 150 mL of YEPG medium (glucose 10 g/L, peptone 5 g/L, yeast extract 2 g/L, MgSO_4_ 0.5 g/L, KH_2_PO_4_ 1 g/L, pH 5.6).

**Figure 1:**
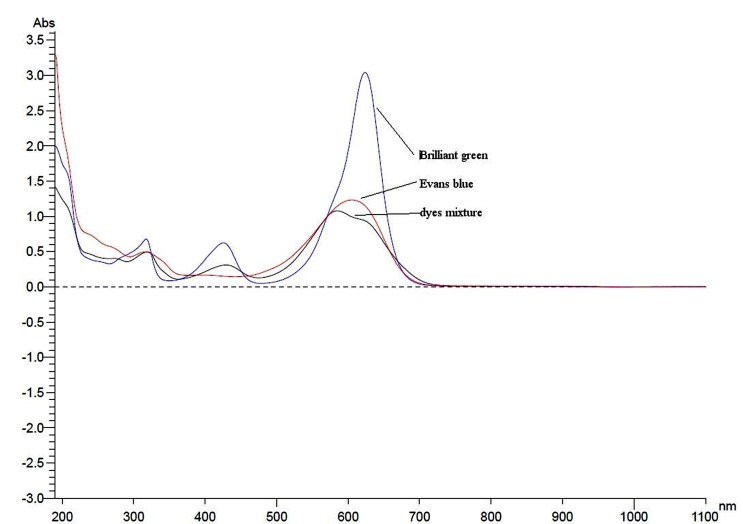
UV-vis scan of the dyes and dye mixture used.

Water solutions of triphenylmethane dye brilliant green (POCh) and diazo dye Evans blue (Sigma-Aldrich) were filter sterilized (Millipore cellulose filters Ø 0.20 μm) and added to 5-day-old fungal cultures. The characteristics of both dyes are presented in Table 1, and the UV-Vis scans are presented in Figure 1. The final concentration of the dye mixture was 0.08 g/L (0.04 g/L of brilliant green and 0.04 g/L of Evans blue). Control samples with the dyes were prepared in sterile medium used for microorganisms cultures and were shaken the same as the inoculated samples. The influence of process conditions (shaking and static cultures) on the decolorization effectiveness was evaluated. As suggested previously by us (Przystas *et al.*, 2012) and other authors (Wesenberg *et al.*, 2003; Kwang-Soo, 2004), some strains remove dyes more effectively under shaking conditions and some under static conditions. Cultures were incubated at 26 °C. Dead biomass was used to estimate biosorption and was obtained by autoclaving (15 min., 121 °C, 1.5 atm) 5-day-old cultures prepared the same as samples with living biomass. All modifications, as well as controls, were performed four times.

**Table 1:**
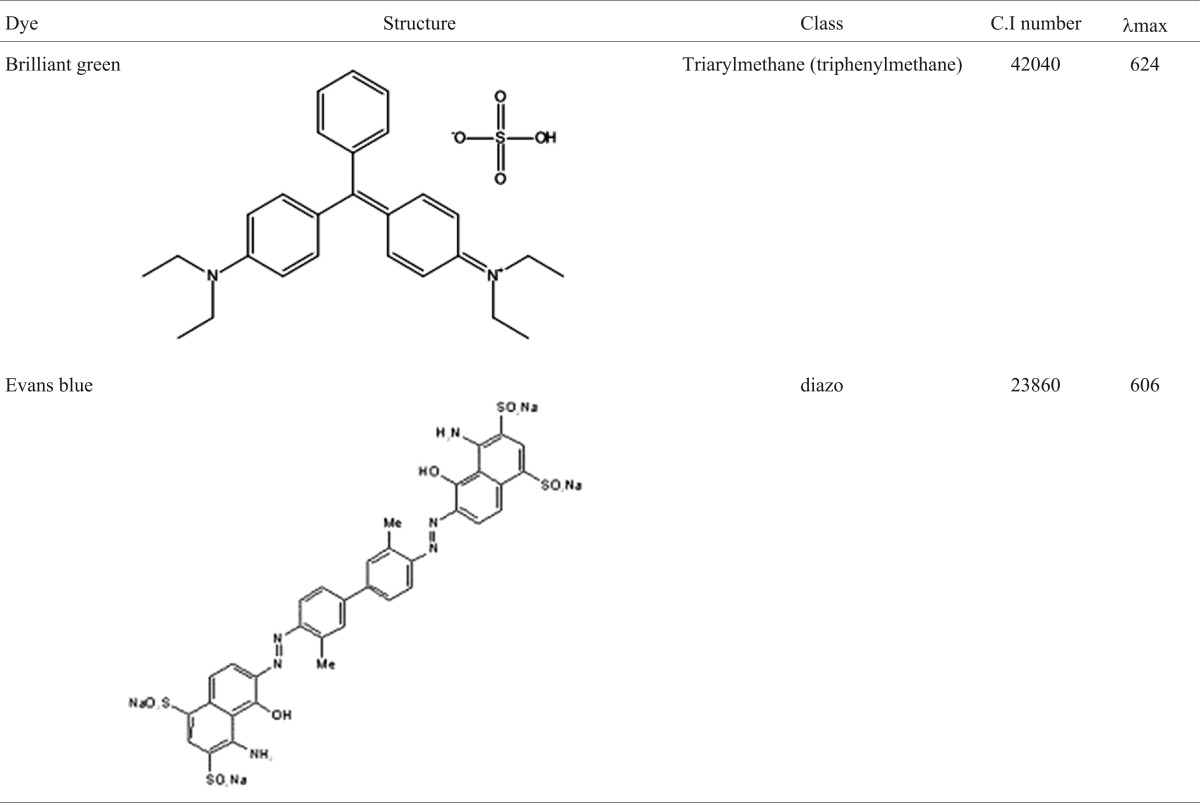
Characteristics of the dyes used.

### Measurement of the decolorization effectiveness and sample toxicity

Absorbance was measured after 1, 6, 24, 48, 72 and 96 h (UV VIS spectrophotometer Hitachi U1900). The dye mixture was examined at three wavelengths (for brilliant green: 624 nm, Evans blue: 606 nm and the dye mixture: 591 nm). All wavelengths were determined experimentally as the wave with maximal absorbance. The percentage of dye removal was calculated according to the formula:

(1) $$\text{R}(\%)=\frac{\text{C}-\text{S}}{\text{C}}\times 100\%$$

where C is the current concentration of dye/dyes in a control sample [mg/L]; S is the current residual concentration of dye/dyes in the samples with fungal biomass [mg/L].

The zootoxicity was evaluated using *Daphnia magna* (OECD 202) and phytotoxicity using an OECD *Lemna* sp. growth inhibition test No. 221. The tests were performed four times. On the base of these results, the acute toxicity units (TU_a_) were calculated, and toxicity classes were established.

(2) $${\text{TU}}_{\text{a}}=\frac{100}{{\text{EC}}_{50}}$$

EC_50_ is the Effective Concentration of a wastewater sample that causes an inhibition of test organisms by 50%. Samples were classified according to ACE 89/BE 2/D3 Final Report Commission EC (TUa < 0.4 - non-toxic (I class); 0.4 ≤ TUa < 1.0 - low toxicity (II class); 1.0 ≤ TUa < 10 - toxic (III class); 10 ≤ TUa ≤ 100 - high toxicity (IV class); TUa > 100 - extremely toxic (V class)).

## Results and Discussion

Structurally different dyes were used for the mixture preparation: Evans blue and brilliant green. Brilliant green is a triphenylmethane dye that has three phenyl groups bound by a central carbon atom. Evans blue is diazo dye characterized by the presence of chromophoric azo groups. Both groups of dyes are used for coloring paper, food, cosmetics, textiles, leather and in medical treatment and analysis (Gill *et al.*, 2001; Robinson *et al.*, 2001; Yesilada *et al.*, 2003; Dos Santos *et al.*, 2007). Because of such wide areas of application, they are often present in surface water.

**Figure 2:**
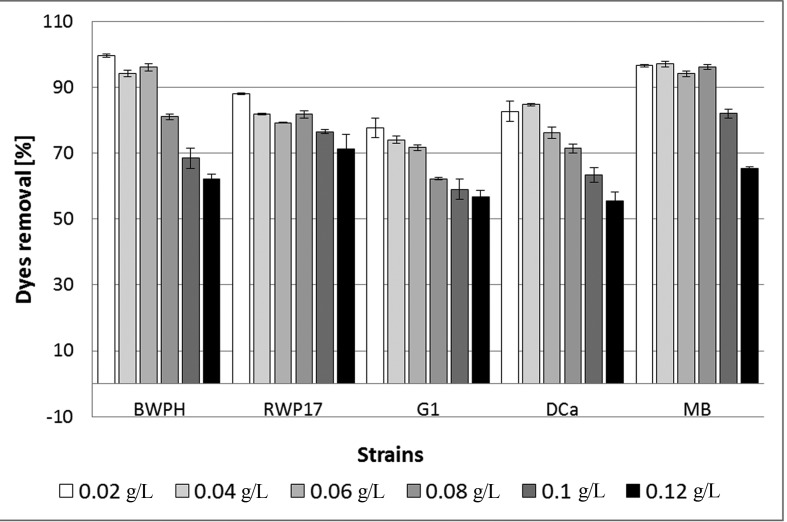
Influence of the dye mixture concentration on the decolorization effectiveness.

Different concentrations of the dye mixture were used to examine the effect of concentration on the decolorization effectiveness. An increase in the dye concentration caused a decrease in the removal effectiveness (Figure 2). Singh and Pakshirajan (2010) tested *Phanerochaete chrysosporium* for the decolorization of single and mixed dyes. As they described, the efficiency of the dye decolorization depended upon their initial concentration. Lower concentrations of the dyes favored easy decolorization. The decolorization efficiency of C.I. Reactive Black 5 by *Debaryomyces polymorphus* was tested by Yang *et al.* (2005). An increase in the dye concentration from 200 mg/L to 400 mg/L decreased the final removal from 100% to 80%. A further increase in the concentration of C.I. Reactive Black 5 (to 1000 mg/L) resulted in a final removal of approximately 30%. Zhuo *et al.* (2011) tested the influence of malachite green, crystal violet, methyl orange and bromophenol blue concentration on the effectiveness of the decolorization. In the case of malachite green and bromophenol blue, an increase in the concentration from 25 mg/L to 200 mg/L had no significant impact on the final dye removal. An increase in the methyl orange concentration from 25 mg/L to 100 mg/L reduced the decolorization efficacy from approximately 100% to less than 60%.

Among the tested fungi, both strains of *Pleurotus ostreatus* (BWPH and MB) were the most effective in the decolorization (Figure 2). They removed more than 60% of the mixture, even at a concentration of 0.12 g/L (62.3% and 65.5%, respectively) and more than 80% of the mixture at a concentration 0.08 g/L (81.1% and 96.2%, respectively). A high effectiveness of decolorization was also reached by strain RWP17 belonging to the species *Polyporus picipes* (81.9% at a concentration of 0.08 g/L and 71.3% at a concentration of 0.12 g/L). A lower effectiveness was reached in samples with strain G1 (56.7% at a concentration of 0.12 g/L and 77.7% at a concentration of 0.02 g/L). Based on these results, for further research, a concentration of 0.08 g/L was chosen; most of the strains removed more than 80% of the dye mixture at this concentration. Dyes used in the mixture were previously tested as a single dye (Przystas *et al.*, 2012) at higher concentrations. As visible in Figure 1, dyes in the mixture interfere. The results of this preliminary test suggest that their toxicity to the tested organisms increased. As proven by Park *et al.* (2007), even small differences in the dye structure, including steric effect and redox potential, may significantly affect the decolorization rate.

**Figure 3:**
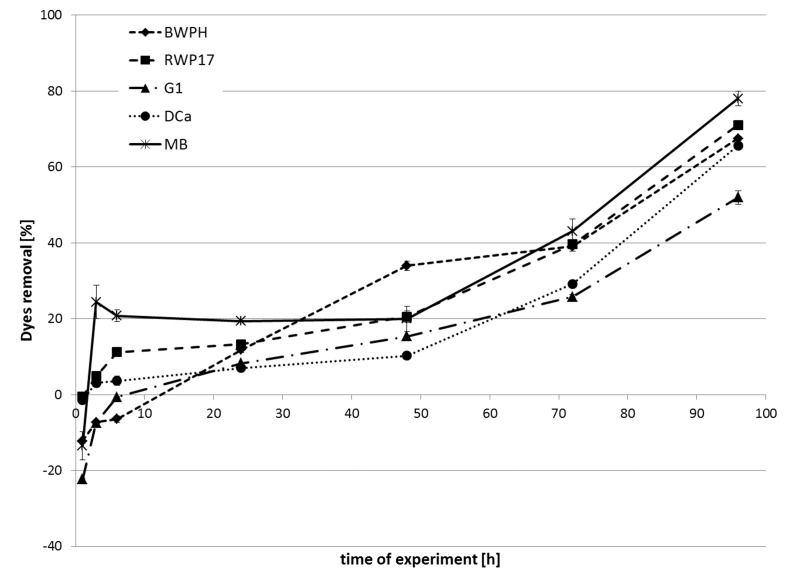
Percentage of removal of the dye mixture at a concentration of 0.08 g/L in static samples.

The results of the main decolorization studies are presented in Table 2 and in Figures 3 [Fig f04]–5. At the beginning of the main experiment, in all samples with strains G1, BWPH and MB, an increase in color was observed. Growth of these strains in media was always connected with changes in the media color from yellow to red. This phenomenon had an influence on the value of absorbance in these samples (absorbance increased in approximately 3–8% of samples with dead biomass and in 10–22.3% of samples with living biomass).

**Table 2:** Results of the decolorization experiment and zoo- and phytotoxicity tests after 96 h of the experiment.

Strains	Modification	BWPH	RWP17	G1	DCa	MB	Controls
% removal of GB after 96 h	shaken biomass	83.58 ± 1.1*	87.13 ± 1.0	79.20 ± 0.6*	84.67 ± 0.6*	90.13 ± 1.7	
	static biomass	67.50 ± 0.5	71.00 ± 0.1	51.93 ± 1.8	65.61 ± 0.1	78 ± 1.9	
	Dead biomass	36.96 ± 0.8*	34.6 ± 0.8	22.3 ± 0.4*	7.02 ± 0.2*	26.4 ± 3.06	
Zootoxicity class in test with *Daphnia magna* (TUa)	shaken biomass	III (8.3)*	III (9.09)	III (4.5)*	III (6.3)*	III (9.09)	V(139.9)*
	static biomass	III (9.25)	III (9.09)	III (9.52)	III (9.09)	III (9.09)	
	Dead biomass	III (8.0)*	III (9.09)	III (2.0)*	III (9.1)*	III (9.09)	
Phytotoxicity class in test with *Lemna minor* (TUa)	shaken biomass	Non-toxic*	Non-toxic	Non-toxic*	III (4.7)*	Non-toxic	IV(83.4)*
	static biomass	I (0.24)	Non-toxic	III (4.97)	III (2.76)	II (0.44)	
	Dead biomass	Non-toxic*	Non-toxic	II (0.9)*	III (8.2)*	Non-toxic	

* Results were published in Przystas *et al.* (2013).

**Figure 4 f04:**
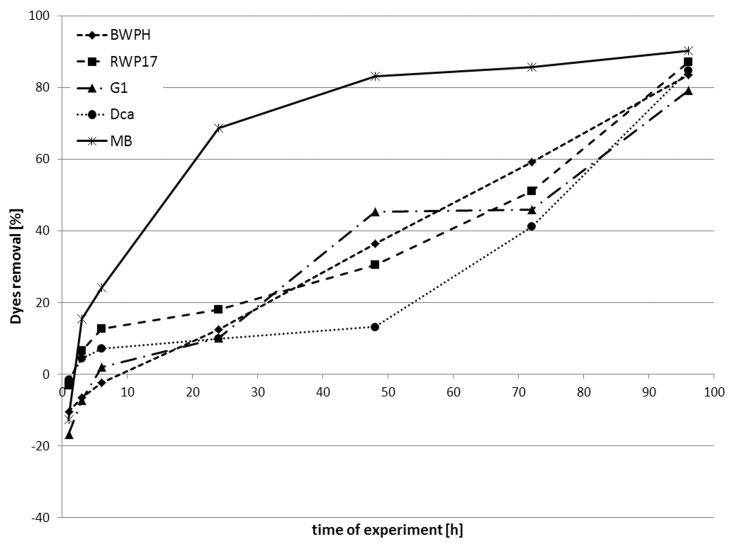
Percentage of removal of the dye mixture at a concentration of 0.08 g/L in shaken samples. (Data for strains BWPH, G1 and DCa were presented previously (Przystas *et al.*, 2013).)

**Figure 5:**
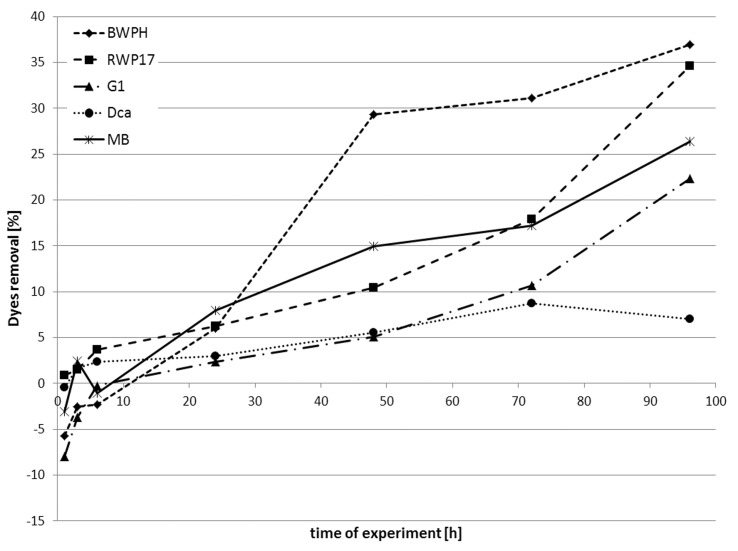
Percentage of removal of the dye mixture at a concentration of 0.08 g/L by the dead biomass of the fungal strains. (Data for strains BWPH, G1 and DCa were presented previously (Przystas *et al.*, 2013).

Among two strains of *P. ostreatus* (MB and BWPH), the most effective was MB, which removed, after 3 h, 15.4% of the dye mixture in shaken samples and 24.4% in static samples. Some fluctuations that were observed for this strain at the beginning of the experiment in static samples were probably connected with adsorption and desorption of the dyes. This result is also confirmed by the results reached using the dead biomass (Figure 5). The decolorization process was faster in shaken than in static samples; after 24 h, removal was 68.6% and 19.4%, respectively. The final decolorization (after 96 h) reached 78% in static and 90.1% in shaken conditions. BWPH removed 11.8% of the mixture in static and 12.5% in shaken samples after 24 h. At the end of the experiment, the results were a bit worse than in the case of strain MB (67.5% and 83.6%, respectively). The results of a previous study (Przystas *et al.*, 2012) showed that single dyes were also better removed in shaken conditions. Brilliant green decolorization was the most effective in samples with strain BWPH and the decolorization of Evans blue in samples with strain MB. Brilliant green was eliminated to 95% (shaken samples) and Evans blue to 89% (static conditions) after 120 h. As presented above, both dyes were used in a previous experiment (Przystas *et al.*, 2012) in higher concentrations (0.06 and 0.15 g/L, respectively) than in the present study (0.08 g/L − 0.04 g/L EB and 0.04 g/L BG).

Experimental conditions also had an influence on the dye mixture removal by strain RWP17. The first changes in sample color were observed after 6 h of the experiment (11.2% removal in static samples and 12.6% in shaken). Slight changes in the color were noted after 24 h (13.3% and 18.4%, respectively). At the end of the experiment, this strain removed 71.0% and 87.1%, respectively, in static and shaken conditions.

The rate of dye mixture removal by strains DCa and G1 was significantly lower than that of other tested samples. After up to 24 h, color reduction was approximately 10%. After 48 h, a significant increase in removal (45.3%) was observed, but only for strain G1 in shaken conditions. The final decolorization effect was 51.9% for strain G1 and 65.6% for DCa in static samples. In shaken samples, it was 79.2% and 84.6%, respectively. Agitation improved the oxygenation of the samples and the contact of the dyes with biomass, which explains why the final results of the decolorization were better. Differences between the results in shaken and static samples were previously reported by Kwang-Soo (2004), Revankar and Lele (2007), Wesenberg *et al.* (2003), Hadibarata *et al.* (2013) and Khan *et al.* (2013). As described by all the above authors, shaking improved the effectiveness of the decolorization. As mentioned by Hadibarata *et al.* (2013), agitation has a positive effect only to an exact level. Agitation that is too strong can limit the decolorization. Wesenberg *et al.* (2003) described the influence of oxygen and agitation conditions on the activity of enzymes. The synthesis and secretion of the ligninolytic enzymes of fungi is often induced by limited nutrient levels (mostly C and/or N) and high oxygen tension. LiP and MnP are especially produced generally at high oxygen tension, but agitation represses this in liquid culture. Another enzyme involved in the process of decolorization, laccase, is produced better in agitated samples. Frequently, more than one isoform of ligninolytic enzymes is expressed by different strains and in culture conditions. This is important to remember during the optimization of fungal decolorization processes (Wesenberg *et al.*, 2003).

The high effectiveness of dye mixture elimination by fungal strains was also described by other authors. Zhuo *et al.* (2011) reached approximately 90% of decolorization of simulated dye effluents within 8 days with strain *Ganoderma* sp. En3. Verma *et al.* (2010) reached the decolorization and detoxification of textile effluents with a mixture of reactive dyes by using 4 strains of marine-derived fungi. The highest decolorization was noted after 6 days for strain NIOCC#C3 (*Pestalotiopsis maculans*). *Bjercandera adusta* was used for the elimination of color of simulated dye effluents by Mohorcic *et al.* (2006). The final decolorization (after 17 days) reached 90%. Additionally, *Geotrichum candidum*, *Trametes versicolor*, *Phanerochaete chrysosporium* and *Schizophyllum commune* were using to decolorize this effluent effectively (60–80%). The decolorization of the dye mixture was also studied by Tilli *et al.* (2011). A few mixtures of dyes belonging to azo and anthraquinonic classes were employed for enzymatic decolorization studies with the extracellular extracts from the white rot fungus *Funalia trogii*. Fungal extracts containing laccase were capable of partially decolorizing dye mixtures. The usage of extracts containing different enzymes resulted in a substantial increase in decolorization for all mixtures of the dyes, which were recalcitrant to the action of laccase alone. All this research confirms that the decolorization effectiveness depends on: the specific structure and composition of the dye, form and composition of the medium, the strain used in the experiment and the site of strain isolation (Dos Santos *et al.*, 2007; Sani and Chand Banerjee, 1999).

Dead biomass of the tested strains removed up to 37% of the dye mixture (Figure 5). The most effective was strain BWPH. Another representative of *P. ostreatus* (strain MB) ultimately adsorbed 26.4%. The lowest adsorption was noted for strain DCa and reached only 7%. Adsorption was first noticeable after 48 h of experiment, and no desorption of the dyes was observed. Park *et al.* (2007) reached approximately 10% of biosorption of reactive orange 16, reactive blue 19, reactive black 5 and acid violet 43 and 40% of acid red by *Funalia trogii*.

To prove the possibility of the application of fungal processes, not only decolorization but also the toxicity analysis of the decolorized products was required. It is difficult to explain why, regardless of the results of the dye removal, in all samples, a decrease in toxicity was noted (Table 2). Zootoxicity of the controls with the dyes was very high, and the samples were classified as extremely toxic. A decrease in toxicity to *Daphnia magna* was observed in all process conditions and for all the strains used, even in samples with dead biomass where the mixture removal was low. After the decolorization, all samples were classified as toxic (III class). Although the samples were classified the same class, differences in the TUa value were observed. Mostly, in shaken samples, the TUa values were lower than in static samples. Controls with the dyes were very toxic (IV class) to *Lemna* sp. In all samples with strain RWP17, after the experiment, no toxicity was observed regardless of the process conditions and effectiveness of the dye removal. In the case of both strains of *P. ostreatus*, shaken samples, as well as samples with dead biomass, were also non-toxic. Static samples were classified to class I (BWPH) or class II (MB) toxicity. The highest phytotoxicity was noted in all samples using strain DCa (class III). As in the case of the zootoxicity, the TUa values in the phytotoxicity tests were also lower in shaken conditions. The toxicity of the stimulated textile effluents was evaluated by Zhuo *et al.* (2011), who proved the toxic impact of effluents on the germination of plant seeds and the growth of shoots and roots. After decolorization by *Ganoderma* sp.En3, the phytotoxicity was lower, the same as in the present study. Apart from the high usefulness of fungal strains in decolorization processes (by biosorption and/or biotransformation), the decrease in the dye toxicity has been frequently proved (Casieri *et al.*, 2008; Przystas *et al.*, 2010; Nascimento *et al.*, 2011; Anastasi *et al.*, 2011; Przystas *et al.*, 2012).

A mixture of dyes belonging to different classes is rarely tested; thus, we concentrated our study on the evaluation of brilliant green (triphenylmethane dye) and Evans blue (azo dye) mixture removal. Such a mixture may be more difficult for the biological treatment than a single dye because interactions between the dyes are observed.

A very high effectiveness of dye removal by living biomass of all the strains used was demonstrated. Up to 90% of the mixture was removed during 96 h of the experiment. Incubation conditions had an influence on the process. Decolorization was the most effective and fastest in shaken samples. The best results were reached for strain MB (representative of *Pleurotus ostreatus* species), which removed more than 90% of the dyes in the shaken samples, as mentioned above. Another representative of *P. ostreatus* (BWPH) removed up to 83.4%, which suggests that the ability for the dye removal is connected to individual features of the strain, not to species. Decolorization was probably connected to biotransformation because dead biomass of the tested fungi absorbed only up to 37% of the dye mixture (strain BWPH). Regardless of the degree of the dye removal, a significant decrease in toxicity was observed, from class V to class III in tests with *D. magna*, and from class IV to non-toxic in tests with *L. minor*. The highest decrease in toxicity was noted in samples with shaken biomass, where the effect on the dye mixture elimination was the best. In summary, our research indicates a very high potential of the tested strains, especially representatives of *Pleurotus ostreatus* and *Polyporus picipes*, to be used for decolorization and detoxification of dye mixtures.
